# Myeloma Bone Disease

**DOI:** 10.1359/jbmr.090901

**Published:** 2009-09-28

**Authors:** Ralph D Sanderson, Joshua Epstein

**Affiliations:** 1 Department of Pathology, Center for Metabolic Bone Disease and Comprehensive Cancer Center, University of Alabama at BirminghamBirmingham, Alabama, USA; 2 Department of Medicine, Myeloma Institute for Research and Therapy, University of Arkansas for Medical SciencesLittle Rock, Arkansas, USA

## INTRODUCTION

Found in >80% of newly diagnosed patients, osteolytic bone disease is the most debilitating manifestations of myeloma.(1) It dramatically impacts patient quality of life through severe pain, fractures, and spinal cord compression, with resulting neurological pathologies. The severity of myeloma bone disease is reflected in the fact that 40–60% of patients suffer from a bone fracture during the course of their disease, higher than that of other major cancers that metastasize to bone (e.g., breast and prostate cancer).(2,3) Bone destruction also releases factors that drive tumor growth further, stimulating disease progression.(4) Thus, it is not surprising that high levels of tumor-induced bone resorption is a strong indicator of poor overall survival of myeloma patients.(5)

Although advances in the understanding of myeloma bone disease have led to promising new therapies, important questions linger. Bone loss in myeloma results from the uncoupling of the mechanisms that control normal bone degradation and bone formation. Overall, myeloma-related bone disease results from the systemic acceleration of bone turnover together with local suppression of osteoblast activity.(6) Early in myeloma progression, there is an elevation of both bone-degrading (osteoclast) and bone-forming (osteoblast) activity. However, as the disease progresses, osteoblast activity is suppressed, shifting the balance to a net increase in bone loss.(7) Although widespread systemic bone loss in myeloma patients occurs, the most dramatic manifestation is the focal lesions that appear as “holes” on X-rays. This results from local suppression of osteoblasts by myeloma cells leaving only osteoclasts, which, in the absence of osteoblast activity, damage bone beyond repair. In contrast, in the small minority of patients that do not exhibit lytic bone disease, the osteoblasts adjacent to myeloma tumor cells remain active. This review focuses on the mechanisms that are responsible for bone loss in myeloma with emphasis on stimulation of osteoclastogenesis and inhibition of osteoblastogenesis. Also included is a brief description of how myeloma bone disease is diagnosed and a summary of current treatments for preserving bone in patients with this cancer.

## HYPERSTIMULATION OF OSTEOCLASTOGENESIS

**FIG. 1:**
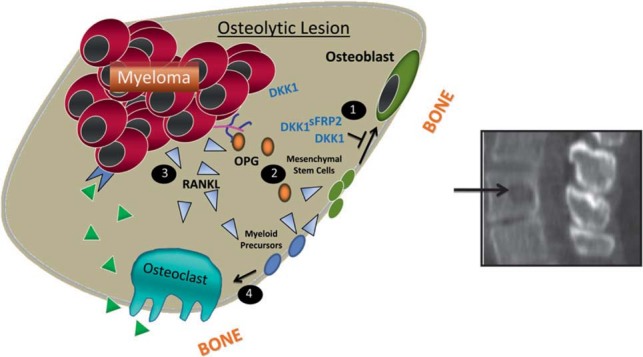
Mechanisms of myeloma-mediated bone destruction. The cartoon depicts a myeloma lesion and the events that occur, leading to formation of an osteolytic lesion. (1) Myeloma cells secrete DKK1 and FRP2 that inhibit Wnt pathway signaling, thus blocking osteoblastogenesis. (2) RANKL (blue triangles) expression on the surface of osteoblasts and bone marrow stromal cells is elevated, and expression of OPG (orange circles) is suppressed. In addition, OPG binds to syndecan-1 on the surface of myeloma cells and is internalized and degraded further shifting the balance toward osteoclastogenesis. (3) Myeloma cells express high levels of either cell surface or soluble RANKL. (4) The high levels of RANKL in the lesion lead to hyperstimulation of myeloid precursor differentiation into osteoclasts. Once mature, the osteoclasts degrade bone and release factors (green triangles) that stimulate myeloma growth. The net effect of these processes is extensive loss of bone at sites of myeloma foci leading to radiologically identifiable osteolytic lesions as shown in the X-ray on the right (arrow points to lesion).

In myeloma, osteoclasts are hyperstimulated predominantly because of dysregulation of three TNF family members: RANK, its ligand (RANKL), and osteoprotegerin (OPG). The binding of RANKL to RANK that is present on the surface of myeloid precursors is needed for differentiation of the precursors into osteoclasts. The activation of RANK is antagonized when soluble OPG binds to RANKL, thereby preventing RANKL activation of RANK. This dampens the rate of osteoclastogenesis. In normal bone, the RANK/RANKL/OPG system works in concert to balance bone turnover and maintain normal homeostasis. In myeloma, both the tumor cells and the bone marrow stromal cells can produce osteoclast-activating factors, thereby shifting the balance toward enhanced osteoclastogenesis and bone destruction. This often occurs within the local tumor microenvironment where osteoclast numbers are seen to be elevated in areas adjacent to myeloma tumor cells. RANKL production is elevated in the bone marrow stroma in myeloma, and in addition, the tumor cells can also express RANKL(8–13) (Fig. 1). High expression levels of membrane-associated RANKL on myeloma cells has been correlated with the presence of multiple bone lesions in myeloma patients.(12,13) In addition, the membrane form of RANKL can be released by proteases. This released, soluble form of RANKL can diffuse away from the local tumor environment to promote widespread osteoclast activation, thereby contributing to systemic bone loss in myeloma.(14) Interestingly, although myeloma cells do not produce OPG, they can diminish the effect that OPG has on inhibiting osteoclastogenesis. This occurs when the heparan sulfate proteoglycan syndecan-1 on the surface of myeloma cells binds to OPG, leading to internalization and degradation of the OPG.(15) Because syndecan-1 is expressed at high levels on most myeloma tumor cells, the binding and degradation of OPG may be a substantial contributor to the bone-degrading phenotype of myeloma. Moreover, clipping of the heparan sulfate chains of syndecan-1 by heparinase is associated with increased syndecan-1 shedding, MMP-9 and VEGF expression, and elevated angiogenesis, events that may also fuel osteolysis.(16–18) This is consistent with a reported positive correlation between MMP-9 and VEGF levels and bone lesion score in myeloma patients.(19)

A number of other effectors have been shown to impact osteolysis including MIP-1α, MIP-β, SDF-1α/CXCR4, IL-3, IL-6, IL-11, and hepatocyte growth factor. Of these, MIP-1α has emerged as a major mediator of myeloma bone destruction. This chemokine is produced by myeloma cells and promotes osteoclastogenesis, and its presence at high levels within the serum of myeloma patients correlates with extensive bone disease and poor survival.(20–23) Gene array profiling indicates that *MIP-1 α* is the gene that is most highly correlated with osteolysis in myeloma patients.(24) Although the mechanisms of MIP-1α-stimulated osteoclastogenesis remain unclear, the finding that RANK knockout mice fail to show enhanced osteoclast formation in calvariae treated with MIP-1α suggests that this chemokine may act through modulation of RANKL.(25) However, another study concluded that MIP-1α increases osteoclastogenesis independently of RANKL.(21) Nonetheless, an important role for MIP-1α in myeloma bone disease has been confirmed by studies in animal models showing that inhibition of MIP-1α expression or blocking its function with antibodies significantly inhibited osteolysis and tumor burden.(26,27)

In addition to osteoclast-mediated destruction of bone, some tumor cells may have the capacity to directly degrade bone. It was discovered many years ago that murine plasmacytoma cells can directly degrade bone in the absence of osteoclasts.(28) More recently, it was shown that some human myeloma cells acquire the functional properties of osteoclasts and degrade bone.(29–31) The ability of tumor cells to directly degrade bone was found to correlate with expression of αvβ3 integrin, and silencing of β3 integrin inhibited the osteolysis in vitro.(32) This suggests that αvβ3 integrin on myeloma cells, in addition to enhancing myeloma cell invasiveness,(33) may also facilitate osteolysis. It will be important to learn more about direct tumor cell destruction of bone to better assess the effectiveness of therapies directed toward inhibiting osteoclast-mediated bone destruction.

Hyperstimulation of osteoclasts in addition to promoting bone destruction also helps drive further tumor progression. Cell-cell contact between myeloma tumor cells and osteoclasts causes release of factors such as IL-6 and osteopontin that support myeloma growth.(9,20) Osteoclasts also release angiogenic factors (including osteopontin) that work in concert with other bone marrow factors to enhance angiogenesis.(34) This link between bone turnover and angiogenesis may be one reason that myeloma presents as such a highly angiogenic disease and may also explain the high rate of relapse and chemoresistance characteristic of myeloma.

## INHIBITION OF OSTEOBLAST DIFFERENTIATION

Bone formation requires proper differentiation of bone marrow mesenchymal stem cells into osteoblasts, a process that is dependent on the canonical wingless (Wnt) signaling pathway.(35) Signaling occurs when Wnt binds to receptors on the cell surface, leading to stabilization of β-catenin in the cell cytoplasm. β-catenin is translocated to the nucleus and stimulates expression of target genes that drive differentiation of the cells into osteoblasts.(36) Enhancement of Wnt signaling by lithium chloride treatment or by overexpression of Wnt3a in bone inhibited bone destruction and reduced tumor burden in a murine models of myeloma.(37,38) Myeloma cells interfere with Wnt-mediated bone formation by secreting DKK-1, a protein that binds to Wnt receptors and competes with Wnt binding to its receptor (Fig. 1). Interestingly, in addition to inhibiting osteoblast differentiation, DKK-1 can also facilitate osteoclastogenesis by enhancing RANKL/RANK and macrophage-colony stimulating factor (M-CSF)/c-Fms interactions.(39,40) Indeed, the ratios of RANKL/OPG in myeloma patients correlate with the extent of bone disease and predict survival.(41,42) DKK-1 is elevated in the bone marrow and blood of myeloma patients with osteolytic lesions.(43) Serum DKK-1 levels correlate with the extent of lytic bone lesions, and patients without bone lesions were found to have lower DKK-1 levels than patients having bone lesions.(44) Beyond these correlative studies, there is mounting evidence that DKK-1 plays a crucial role in regulating bone loss in vivo. For example, transgenic mice overexpressing DKK-1 exhibit osteopenia, whereas reduction of DKK-1 expression increases bone mass.(45,46) Also, antibodies to DKK-1 have been shown to reduce osteolytic lesions and tumor burden in animal models of myeloma, thereby confirming the important negative regulatory effect that DKK-1 has on osteoblastogenesis.(47,48)

In addition to DKK-1, there is evidence that myeloma cells also secrete another soluble Wnt inhibitor, frizzled-related protein-2 (FRP-2), that may play a role in dysregulation of osteoblast differentiation.(49) Secretion of soluble FRP-2 (sFRP-2) was often found in patients having advanced bone lesions. In vitro, exogenous sFRP-2 suppresses BMP-2-induced osteoblast differentiation, whereas immunodepletion of sFRP-2 restores mineralized nodule formation by osteoblasts.(49)

## DIAGNOSIS

As mentioned above, lytic bone disease in myeloma can be systemic (general osteopenia) and focal. Whereas biochemical markers have been suggested as diagnostic parameters of myeloma-associated osteolysis,(50–55) these cannot differentiate between focal and systemic bone loss. Systemic bone loss can be diagnosed with bone densitometry measurements or even routine metastatic bone surveys (MBSs). The standard of diagnosis of myeloma bone disease is by means of radiography. Focal osteolytic lesions develop in areas of focal myeloma growth, which are visible on MRI even before the identification of focal osteolysis by standard MBS.(56) Standard MBS has the low sensitivity of standard x-radiographs, which require loss of at least 30% of the bone matrix before osteolysis can be recognized. In contrast, helical CT, because of its superior contrast resolution, has the sensitivity to identify focal bone loss in the majority of cases where MBS failed.(56) CT is thus the modality of choice to diagnose focal bone loss in myeloma.(57–59)

## TREATMENT

Myeloma-associated osteolytic lesions do not repair, even in patients in complete remission for many years. The bisphosphonates, which continue to be the standard of care for the treatment of myeloma bone disease, prevent further bone loss by inhibiting osteoclast activity, but these decrease skeletal related events by only 50%. In addition, 4–6% of patients receiving bisphosphonates develop osteonecrosis of the jaw, and often treatment has to be stopped because of kidney toxicity.(4,60) Thus, treatments with better efficacy and fewer side effects than bisphosphonates are needed.

The proteasome inhibitors, particularly bortezomib (Velcade), have emerged as important bone-preserving agents in myeloma. Although introduced as a therapeutic strategy to inhibit myeloma growth, bortezomib has an important positive impact on bone, and this might contribute to the overall antimyeloma affect of the compound. Bortezomib was shown to reduce tumor burden and enhance BMD in an animal model of myeloma(61) and to promote osteoblast differentiation through Wnt-independent activation of β-catenin.(62) In clinical studies of relapsed myeloma patients, bortezomib was shown to reduce DKK-1 and RANKL levels, reflecting its potential for mediating important bone protective effects even in advanced disease.(63) Proteasome inhibitors can have important anabolic effects on bone as supported by patients that show an increase in markers of osteoblast activity such as alkaline phosphatase and osteocalcin.(64–66) A recent report showed that the hydroxamate-based histone deacetylase inhibitor JNJ-26481585, when used in combination with bortezomib, significantly reduced tumor burden, angiogenesis, and myeloma bone disease, including a pronounced reduction of osteoclasts and increase of osteoblasts, trabecular bone volume, and trabecular number.(67) This underscores the need to explore bortezomib in combination with various anticancer therapies to find the best therapeutic approach for preserving and building bone.

Another promising approach for treating myeloma bone disease is anti-RANKL therapy using the humanized monoclonal antibody denosumab. This antibody is highly specific for RANKL and effectively blocks the interaction of RANKL with RANK, thereby diminishing osteoclastogenesis. A phase I clinical trial of denosumab in myeloma patients indicated that the antibody suppresses bone resorption without obvious negative side effects.(68) Further studies that include large numbers patients and longer follow-up are needed to determine the usefulness of this antibody against myeloma bone disease.

As described above, DKK1 is central to myeloma bone disease. The lack of repair of myeloma-induced osteolytic lesions likely reflects the continued secretion of DKK1 by dormant myeloma cells in focal lesions, even in patients in complete remission and after resolution of MRI focal lesions.(56) Inhibition of Dkk1 by monoclonal antibody effectively increased osteoblast numbers and activity in mouse models of myeloma, increased BMD of mouse bones and human bone implants, and reduced tumor burden in most experiments.(47,48,69) These results raise hope for a treatment that will repair myeloma osteolytic lesions. Antibodies against DKK1 are currently in early clinical trials. Regarding repair of osteolytic lesions, there is also potential for use of mesenchymal stem cells. These cells, when injected into bone, have been shown to inhibit myeloma tumor growth and increase BMD in an animal model.(70) This type of cell-based therapy could be of great value to treat myeloma patients, particularly if the cells could be engineered to home specifically to myeloma bone lesions after intravenous delivery.

## SUMMARY

Bone disease is a characteristic feature of myeloma and causes devastating side effects that impact patient quality of life and survival. New findings over the last several years have led to a better understanding of the molecular mechanisms regulating bone disease including the understanding that myeloma bone disease results from hyperstimulation of osteoclastogenesis and inhibition of osteoblastogenesis. From these mechanistic findings, promising new therapies including bortezomib and denosumab have evolved and give hope for lessening the impact of osteolytic bone disease in myeloma. New therapies such as anti-DKK1 antibodies are on the horizon. Despite these advances, lingering questions remain that need to be addressed. Why in a small minority of patients do osteoblasts near tumor foci continue to survive and proliferate, whereas in most patients osteoblastogenesis is inhibited? Can combinations of bone preserving and bone forming therapies lead to repair of osteolytic lesions in myeloma? To what extent do myeloma tumor cells exhibit direct osteolytic effects separate from osteoclasts, and how can these effects be diminished? Tackling these and other remaining mechanistic questions will lead to even better therapeutic approaches to controlling myeloma bone disease.
